# Species-Specific Regulation of TRPM2 by PI(4,5)P_2_ via the Membrane Interfacial Cavity

**DOI:** 10.3390/ijms22094637

**Published:** 2021-04-28

**Authors:** Daniel Barth, Andreas Lückhoff, Frank J. P. Kühn

**Affiliations:** Institute of Physiology, Medical Faculty, RWTH Aachen University Hospital, D52057 Aachen, Germany; a.lueckhoff@physiologie.rwth-aachen.de (A.L.); fkuehn@ukaachen.de (F.J.P.K.)

**Keywords:** TRPM2, PIP2, phospholipid, phosphoinositides, patch clamp

## Abstract

The human apoptosis channel TRPM2 is stimulated by intracellular ADR-ribose and calcium. Recent studies show pronounced species-specific activation mechanisms. Our aim was to analyse the functional effect of phosphatidylinositol 4,5-bisphosphate (PI(4,5)P_2_), commonly referred to as PIP_2_, on different TRPM2 orthologues. Moreover, we wished to identify the interaction site between TRPM2 and PIP_2_. We demonstrate a crucial role of PIP_2_, in the activation of TRPM2 orthologues of man, zebrafish, and sea anemone. Utilizing inside-out patch clamp recordings of HEK-293 cells transfected with TRPM2, differential effects of PIP_2_ that were dependent on the species variant became apparent. While depletion of PIP_2_ via polylysine uniformly caused complete inactivation of TRPM2, restoration of channel activity by artificial PIP_2_ differed widely. Human TRPM2 was the least sensitive species variant, making it the most susceptible one for regulation by changes in intramembranous PIP_2_ content. Furthermore, mutations of highly conserved positively charged amino acid residues in the membrane interfacial cavity reduced the PIP_2_ sensitivity in all three TRPM2 orthologues to varying degrees. We conclude that the membrane interfacial cavity acts as a uniform PIP_2_ binding site of TRPM2, facilitating channel activation in the presence of ADPR and Ca^2+^ in a species-specific manner.

## 1. Introduction

Transient receptor potential melastatin type 2 (TRPM2) is a non-selective cation channel that belongs to the TRPM subfamily, consisting of TRPM1 to TRPM8. Extensive cryo-EM studies in the past few years have revealed the complex structure of TRPM2 (reviewed in [1]). As a result, the channel was identified as a homotetramer with each monomer consisting of an N-terminal TRP melastatin homology region (MHR) formed by four subdomains (MHR1-4). The subsequent transmembrane region (TM) is composed of the S1–S4 voltage sensor-like domain followed by the pore-forming S5–S6 domain. Right after the TM region are the TRP helices, a rib helix, and a pole helix with the adjacent NUDT9 homology (NUDT9H) domain at the C-terminus.

The principal stimulus for gating of TRPM2 channels is ADP-ribose (ADPR) which is produced in many cells during oxidative stress [2,3]. Moreover, intracellular calcium is an important co-agonist, leading to self-enhancing processes during TRPM2 activation because the channel enables calcium influx [4,5]. Therefore, TRPM2 takes part in many physiological processes such as immune response [6], oxidative stress-induced apoptosis [7], insulin secretion [8,9], and temperature regulation [10].

Recently, functional analyses of species variants as well as several cryo-EM studies have revealed elementary species-specific differences in the ADPR-dependent gating mechanism of TRPM2 (reviewed in [1]). The presence of an additional ADPR binding pocket beside the classical one in the NUDT9H domain is now experimentally confirmed [11,12,13,14,15,16]. This dualism in ADPR binding is present in all channel orthologues investigated so far, but the functional importance of the two binding pockets is quite different depending on the species variant. In TRPM2 of the sea anemone (nvTRPM2) an N-terminal ADPR binding pocket (NUDT9H) exclusively has ADPRase function [11,13,16,17]. In contrast, in hsTRPM2 and drTRPM2 the presence of the NUDT9H domain is essential for channel function whilst significant ADPRase activity is missing [3,17,18,19,20,21]. Moreover, in hsTRPM2 binding of ADPR to both binding pockets seems to be required for channel gating [14], while in drTRPM2 binding of ADPR to NUDT9H is rather unimportant [13,22]. Thus, it is not entirely clear whether the crucial role of the NUDT9H domain in vertebrate TRPM2 derives from direct ADPR binding or solely from the facilitation of interdomain interactions [13,14,15,22], that are absent in non-vertebrate orthologues [12,16,17]. Differences in how ADPR stimulates TRPM2 channels in different species are not confined to the intramolecular mechanisms of how gating is achieved but extend to the potency of ADPR. Considerably higher ADPR concentrations are required in hsTRPM2 compared with the other two orthologues [3,13,23]. Due to these species-specific gating mechanisms of TRPM2, it is of particular interest to study different TRPM2 orthologues with regard to various TRPM2 modulators to understand the full scope of channel activation. 

In the past decades, a number of phospholipids, particularly phosphatidylinositol 4,5-bisphosphate (PI(4,5P_2_), referred to as PIP_2_, have been shown to regulate the activity of a vast variety of ion channels and transport proteins [24]. This observation was first reported by Choquette et al., in 1984 via a study investigating brain Ca^2+^-ATPases in liposomes in the presence and absence of PIP_2_. They found that PIP_2_ stimulates the activity of the Ca^2+^-ATPases [25]. Since then, several other ion channels were identified to be regulated by PIP_2_ including members of the TRP family. Whilst some channels, including TRPM4, TRPM5, TRPM8, TRPV5, and TRPV6 are positively regulated by PIP_2_ [26], others are negatively regulated including TRPC4, TRPP2, and TRPP3 [27,28,29]. In 2012, the human species variant of TRPM2 was first shown to be positively regulated by PIP_2_ when expressed in *Xenopus laevis* oocytes, where PIP_2_ was proposed to regulate the Ca^2+^ sensitivity of TRPM2 activation [30]. This positive regulation by PIP_2_ is also present in nvTRPM2 [31]. While the PIP_2_-interaction sites for TRPM2 have not yet been identified, they are discovered for their closest relative within the TRP family, TRPM8. In TRPM8, mutation of conserved positively charged amino acids within the membrane interfacial cavity reduces the sensitivity of the channel for PIP_2_ and increases inhibition by PIP_2_ depletion [27,32,33]. The membrane interfacial cavity is formed by the pre-S1 domain, the junction between S4 and S5, TRP domain, and MHR4 from the adjacent subunit. Since these domains and residues are highly conserved within the TRPM family and similar mutations in TRPM5 result in reduced PIP_2_ induced currents, it is thought that PIP_2_ interaction with TRPM2 is facilitated in a similar fashion.

Therefore, the aim of the study was a comprehensive analysis of the effect of PIP_2_ on TRPM2 activity. Specifically, we focused on the PIP_2_ binding site in TRPM2 and potential species-dependent differences due to the unique species-specific gating mechanism. These experiments should lead to a better understanding of how TRPM2 activity is modulated by PIP_2_ and may provide a new aspect of TRPM2 regulation in physiological processes.

Here, we show that three species variants of TRPM2, representing a broad spectrum of metazoan evolution, are positively regulated by PIP_2_ when expressed in HEK 293 cells. The comparative analysis shows, however, that the extent of the positive regulation by PIP_2_ differs depending on the species variant. In addition, this study provides the first experimental evidence for a uniform interaction site between TRPM2 and PIP_2_ which is similar to the membrane interfacial cavity previously described for TRPM8 [32].

## 2. Results

### 2.1. PIP_2_ Is a Necessary Cofactor for TRPM2 Activation in Mammalian Cells

To study the effects of PIP_2_ on TRPM2 channels, inside-out patch clamp experiments were performed on different species variants of TRPM2 heterologously expressed in HEK 293 cells: Human TRPM2 (hsTRPM2), zebrafish (*Danio rerio*) TRPM2 (drTRPM2), and sea anemone (*Nematostella vectensis*) TRPM2 (nvTRPM2). Expression was so high that macroscopic currents rather than single-channel events were consistently observed after stimulation. PIP_2_ and other PIP analogues were used in their synthetic diC8-form which are commercially available and sufficiently water-soluble and applied to the cytosolic side of the membrane of inside-out patches by a multi-barrel perfusion system.

**Figure 1:**
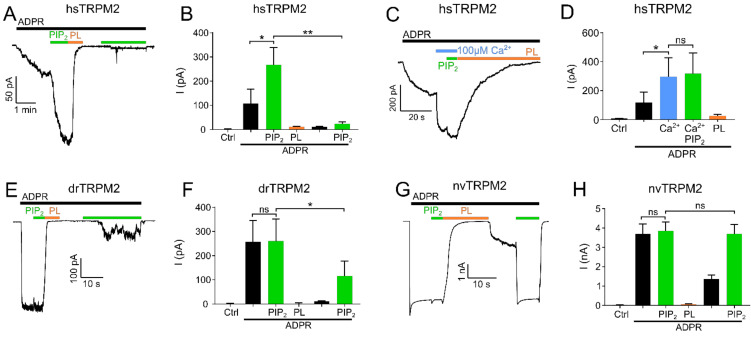
PIP_2_ is a necessary cofactor for TRPM2 activation. Inside-out patch clamp recordings of HEK-293 cells expressing TRPM2 at −60 mV and 1 µM Ca^2+^ unless stated otherwise. Currents were activated by cytosolic exposure to ADPR (black bar). (**A**) Representative current trace of hsTRPM2 activated by cytosolic exposure to 300 µM ADPR (black bar) and 25 µM PIP_2_ (green bar) and blocked by 15 µg/mL polylysine (PL, orange bar). (**B**) Summary of the effects of ADPR, PIP_2,_ and polylysine on hsTRPM2 currents. Statistical analysis reveals a significant current increase via PIP_2_ after the initial ADPR induced current plateaued. The current was abolished upon exposure to polylysine and unable to recover via a second PIP_2_ application. Control (Ctrl) represents standard bath solution in absence of ADPR. (**C**,**D**) Representative current trace and statistics of the PIP_2_ effect following elevated intracellular Ca^2+^ concentrations (100 µM) for hsTRPM2. Application of 100 µM Ca^2+^ causes a strong current increase in hsTRPM2 which is only slightly further increased upon external PIP_2_ addition. Current traces of drTRPM2 (**E**) and nvTRPM2 (**G**) exposed to 100 µM ADPR, 25 µM PIP_2_ and 15 µg/mL polylysine. Statistical analysis shows no significant difference for drTRPM2 (**F**) and nvTRPM2 (**H**) between the current observed during exposure to ADPR and the additional application of PIP_2_. (**F**) Fractional reactivation of drTRPM2 currents by second PIP_2_ application after the current was abolished via polylysine while nvTRPM2 (**H**) fully recovered. Data are presented as mean ± SEM analysed via one-way ANOVA with Dunn’s multiple comparison test; *n* = 3–8; * *p* < 0.05, ** *p* < 0.01, ns = not significant.

**Figure 2:**
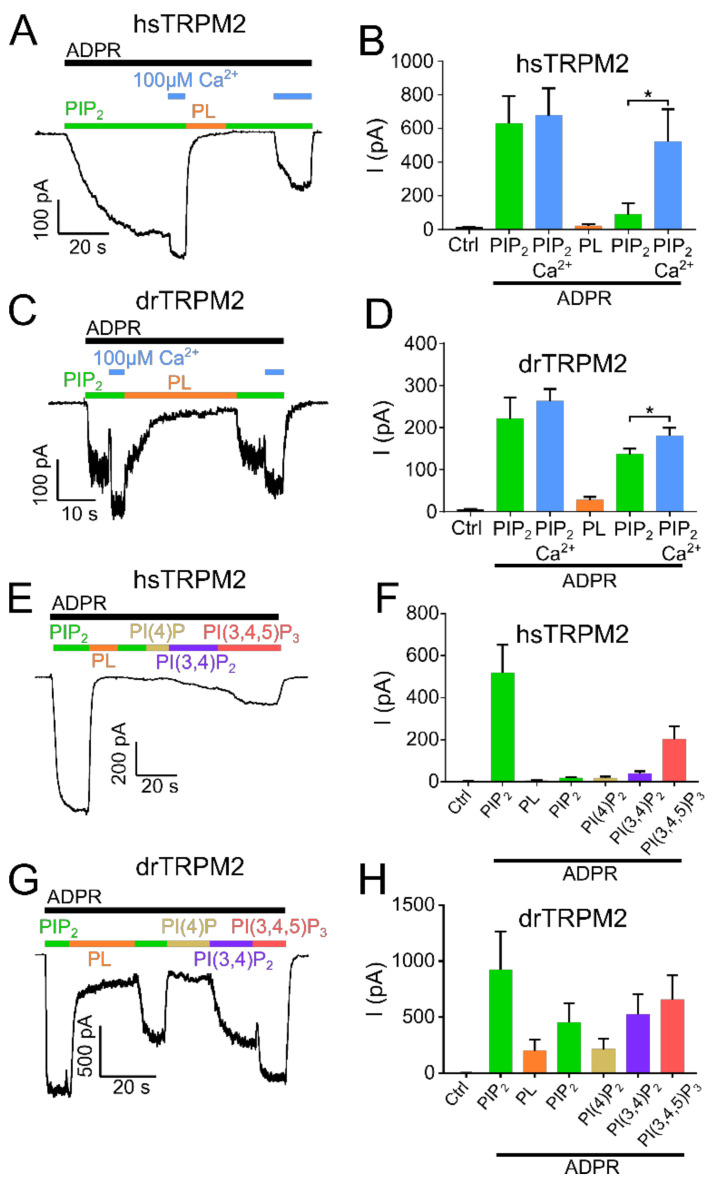
Influence of high calcium and PIP_2_ derivatives on TRPM2 current restoration. Recordings of HEK-293 cells expressing TRPM2 at −60 mV in the inside-out patch clamp configuration. Currents were activated by cytosolic exposure to ADPR (black bar) and PIP_2_ (25 µM) (green bar). Representative current trace and statistics of hsTRPM2 (**A**,**B**) and drTRPM2 (**C**,**D**) at high Ca^2+^ concentrations (100 µM). High Ca^2+^ in presence of PIP_2_ and ADPR (blue bar) causes current restoration after the current was inhibited via 15 µg/mL polylysine (PL, orange bar). Representative recordings and summary of current statistics of the effect of PI derivatives on hsTRPM2 (**E**,**F**) and drTRPM2 (**G**,**H**) currents using 1 µM cytosolic Ca^2+^. Statistical analysis was performed via paired Student’s *t*-test and data presented as mean ± SEM; *n* = 4–7; * *p* < 0.05.

In hsTRPM2, no currents were observed in the absence of ADPR but the continuous application of ADPR (300 µM) resulted in gradually developing, long-lasting macroscopic currents (Figure 1A,B). When a plateau was reached after approximately 2 min, PIP_2_ (25 µM) was applied in addition to ADPR. This increased the current significantly by about 2.5-fold. Interestingly, the strongly increasing intracellular concentration of Ca^2+^ (100 µM) prior to PIP_2_ application caused a significant current increase that was only marginally further increased by the addition of 25 µM PIP_2_ (Figure 1C,D). This result shows that when the applied concentrations of Ca^2+^ are too low, the starting open probability of hsTRPM2 is also low, and therefore can be further increased by PIP_2_ application. Following PIP_2_ addition, a subsequent switch to a perfusion solution without PIP_2_ but with ADPR and polylysine (15 µg/mL) completely and rapidly abolished the currents. Polylysine acts as a PIP_2_ scavenger and therefore inhibits the activity of PIP_2_ activated ion channels [34,35]. Washout of polylysine, under the continuing application of ADPR, failed to restore any currents. The current rescue was also not achieved with the subsequent application of PIP_2_ (25 µM), inducing either negligible or no response (Figure 1A,B) even with PIP_2_ concentrations up to 100 µM (Supplementary Figure S1A). Only when Ca^2+^ (100 µM) was supplemented to PIP_2_, a partial rescue of the current was achieved (Figure 2A,B). This current restoration was PIP_2_ and calcium-dependent as high calcium concentrations in the absence of PIP_2_ were not sufficient to restore currents (Supplementary Figure S1C,D). We next tested whether other PI derivatives, PI(4)P, PI(3,4)P_2_, and PI(3,4,5)P_3_, might be more effective than PIP_2_ on the restoration of polylysine-abolished currents. Only for PI(3,4,5)P_3_ was such a tendency observed (Figure 2E,F). As hsTRPM2 and drTRPM2 currents also show rundown in HEK-293 cells (Supplementary Figure S2B,C), although in a strongly delayed manner, if compared to the results obtained from Xenopus oocytes [17,30] while nvTRPM2 currents do not show any rundown at all (Supplementary Figure S2D), current restoration upon polylysine treatment could be affected by this process. However, over the time course of our recordings rundown should only marginally affect current restoration.

Examining drTRPM2, a concentration of 100 µM ADPR (please refer to Supplementary Figure S2A for ADPR dose-response recordings of hs-, dr- and nvTRPM2) was sufficient to evoke strong and rapid increases in currents but the additional application of PIP_2_ did not increase the ADPR-induced current further, suggesting that drTRPM2 may already be saturated with the pre-existing PIP_2_ concentration in the patch (Figure 1E,F). In addition, when compared to hsTRPM2 starting open probability of drTRPM2 at 1 µM calcium may be already close to the maximum, resulting in no further current increase during PIP_2_ application. Similar to hsTRPM2, polylysine caused a complete current decay. Subsequent washout of polylysine only led to minuscule currents whereas the additional application of PIP_2_ rescued about half of the initial current amplitudes (Figure 1E,F). In addition, increasing the Ca^2+^ concentration to 100 µM significantly increases current restoration via PIP_2_ (Figure 2C,D), even though this effect is less pronounced compared with hsTRPM2. In contrast to hsTRPM2, minor currents were restored via high calcium concentrations (100 µM) in absence of PIP_2_ (Supplementary Figure S1E,F). Additionally, application of the PIP_2_ derivatives PI(3,4)P_2_ and PI(3,4,5)P_3_ tends to improve current restoration while PI(4)P has no effect (Figure 2G,H).

In nvTRPM2, ADPR (100 µM) caused a rapid current increase which was not further enhanced by PIP_2_ (Figure 1G,H). Again, similar to drTRPM2 this may be caused by an increased starting open probability at 1 µM Ca^2+^ compared with hsTRPM2. Once more, polylysine caused the current to decay completely. In contrast to the findings on the other two orthologues, washout of polylysine led to a moderate current increase, and the current was completely restored to the initial maxima by the second addition of PIP_2_. Therefore, nvTRPM2 is the only TRPM2 species tested in this study that was able to fully recover the initial current via PIP_2_ after polylysine was used to scavenge PIP_2_. Our data suggest that even though all three TRPM2 species variants are positively regulated via PIP_2_, there are significant differences in the way PIP_2_ addition or withdrawal affects channel activity in the different species variants.

### 2.2. PIP_2_ Sensitivity Varies among TRPM2 Species

**Figure 3:**
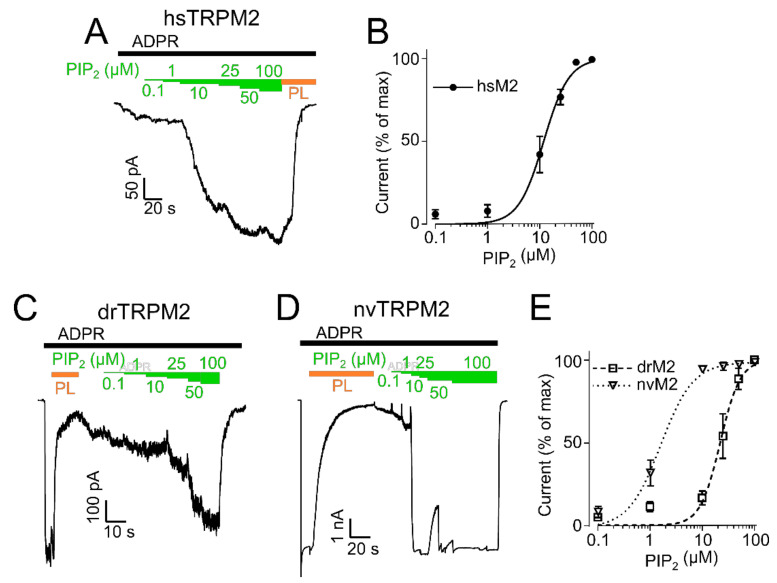
PIP_2_ sensitivity of TRPM2. PIP_2_ dose-response recordings of inside-out patches of HEK-293 cells expressing hsTRPM2 (**A**), drTRPM2 (**C**), and nvTRPM2 (**D**). ADPR (black bar) was used to initiate TRPM2 currents followed by increasing concentrations of PIP_2_ (green bars). Polylysine (PL, orange bar) was utilized to scavenge PIP_2_. Summary of the PIP_2_ dose-response recordings calculated as a percentage of the maximum current obtained at 100 µM PIP_2_ (hsTRPM2 (**B**), drTRPM2, and nvTRPM2 (**E**)). Data were analysed from five to six independent experiments.

Since the initial experiments indicated species-related differences in the sensitivity of TRPM2 orthologues regulated by PIP_2_, we decided to induce gradual responses by various PIP_2_ concentrations to determine EC_50_ values. After hsTRPM2 stimulated with ADPR evoked a stable baseline current, increasing PIP_2_ concentrations (from 0.1 to 100 µM) were consecutively added (Figure 3A). An EC_50_ value of 11.9 ± 1.1 µM (Figure 3B) was determined for hsTRPM2. For drTRPM2 and nvTRPM2, a different protocol had to be applied since ADPR (100 µM) in absence of external PIP_2_ already elicited maximal currents that were not further enhanced by additional PIP_2_. Hence, polylysine was used to scavenge PIP_2_ from ADPR-stimulated patches prior to the PIP_2_ dose-response. Then, PIP_2_ was gradually replenished through the perfusion system (Figure 3C for drTRPM2 and Figure 3D for nvTRPM2). The EC_50_ values calculated in this protocol were 22.3 ± 2.0 µM for drTRPM2 and 2.1 ± 0.2 µM for nvTRPM2 (Figure 3E), however, slow time-dependent current recovery via endogenous free PIP_2_ may contaminate the effects marginally. These data indicate higher PIP_2_ sensitivity for nvTRPM2 than for drTRPM2. It should be noted that a direct quantitative comparison of drTRPM2 and nvTRPM2 with hsTRPM2 was not possible because the EC_50_ values were determined under different conditions. However, hsTRPM2 seems to be the least sensitive towards PIP_2_ which was also evident as washout of externally applied PIP_2_ caused a reduction in hsTRPM2 current (Supplementary Figure S1G). The respective EC_50_ values are the basis for the determination of how various mutations affect the PIP_2_ sensitivity of each orthologue.

### 2.3. Residues within the Membrane Interfacial Cavity Are Putative PIP_2_-Interaction Sites

**Figure 4:**
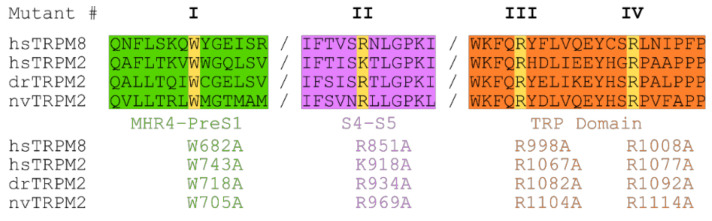
Sequence alignment of TRPM2 and TRPM8. Sequence alignment of the membrane interfacial cavity (formed by: Pre-S1 domain and MHR4 from the adjacent subunit (green), junction between S4 and S5 (purple) and TRP domain (orange)) of hs-, dr- and nvTRPM2 and hsTRPM8. Target positions (yellow) within MHR4-PreS1 labelled mutant I (hsW743, drW718, and nvW705), S4–S5 linker labelled mutant II (hsK918, drR934, and nvR969) and TRP domain separated in mutant III (hsR1067, drR1082, nvR1104) and mutant IV (hsR1077, drR1092, nvR1114).

Next, we aimed to identify potential PIP_2_ interaction sites and their contribution to PIP_2_-dependent channel regulation. In the cation channel TRPM8, the closest relative of TRPM2, several amino acid residues have been associated with such a role [27,32,33]. Most of them are positively charged and located within the membrane interfacial cavity which in turn is formed by the pre-S1 domain, the junction between S4 and S5, the TRP domain, and MHR4 from the adjacent subunit. In analogy, we chose four residues most critical in TRPM8 and performed a substitution with alanine at corresponding positions of each of the three species variants of TRPM2. Figure 4 specifies the exact position of each of these point mutations which we designate as mutations I to IV for each species for simplicity (e.g., homologous exchange of W743A in hsTRPM2, W718A in drTRPM2 and W705A in nvTRPM2 are all referred to as mutation I).

**Figure 5:**
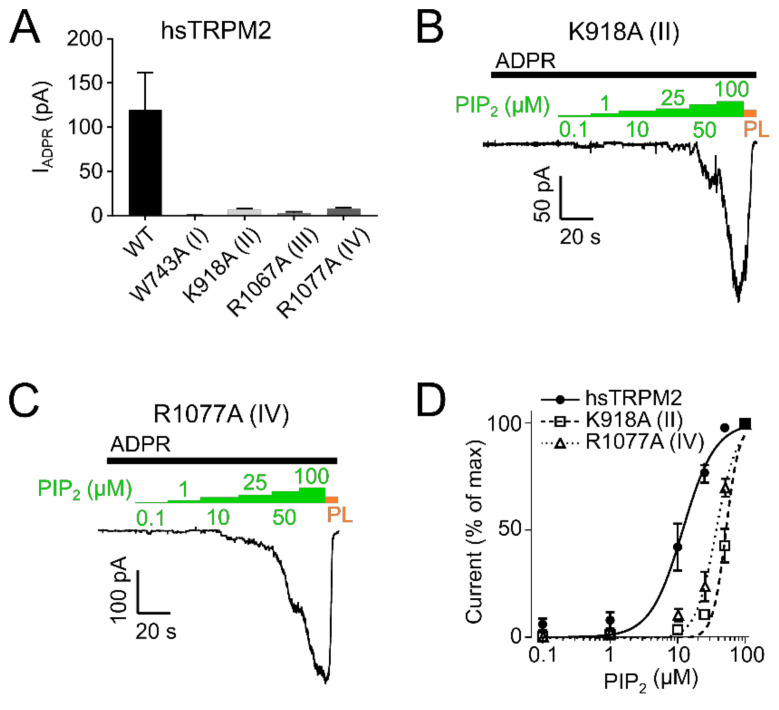
PIP_2_ sensitivity of hsTRPM2 membrane interfacial cavity mutant channels. (**A**) Current amplitudes of wild-type and mutant hsTRPM2 channels in response to 300 µM ADPR. Representative current trace of K918A (II) (**B**) and R1077A (IV) (**C**) exposed to increasing PIP_2_ (green bar) concentrations in the presence of 300 µM ADPR (black bar). 15 µg/mL polylysine (PL, orange bar) was used to block PIP_2_ mediated currents. (**D**) Data summary of PIP_2_ dose-response experiments showing a clear right shift of mutants K918A (II) and R1077A (IV) compared with the wild-type. Data presented as mean ± SEM; *n* = 5.

In hsTRPM2 we found that W743A (I) showed a complete absence of ADPR induced currents and was therefore considered non-functional (Figure 5A). Analysis of R1067A (III) showed that ADPR induced either negligible or no response (Figure 5A) and was also unaffected by additional application of up to 100 µM PIP_2_ (Figure S1B), thereby preventing a quantitative analysis of the PIP_2_ effects. K918A (II) and R1077A (IV) showed substantial decreases in the current amplitudes (Figure 5A).

**Figure 6:**
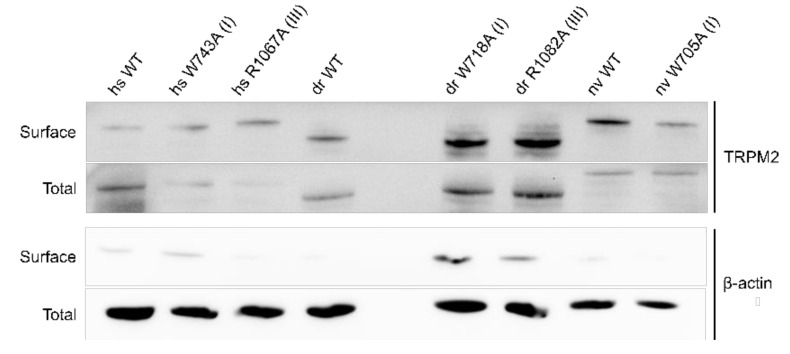
Biotinylation assay of TRPM2 expressed in HEK-293 cells. Representative surface and total expression of hs-, dr- and nvTRPM2 WT and point mutations via biotinylation. β-actin was used as the loading control.

As a control, membrane expression was tested with biotinylation assays showing no significant differences in membrane expression between hsTRPM2 WT and mutants (Figure 6).

**Figure 7:**
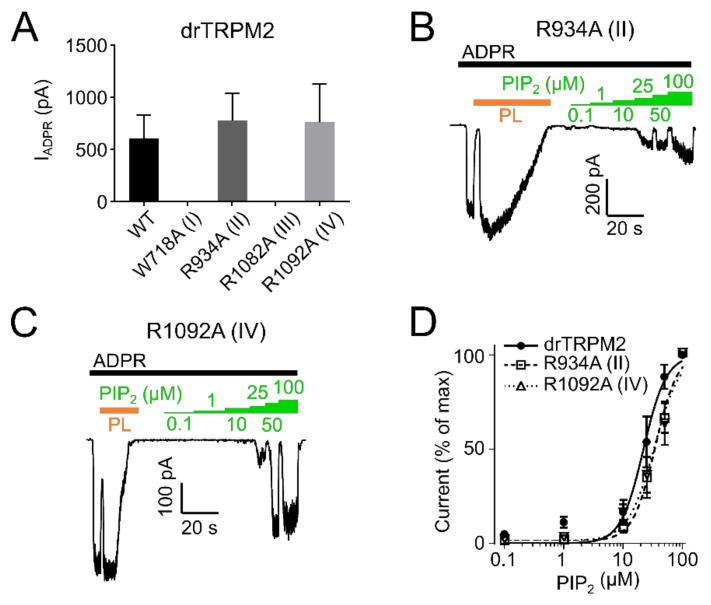
PIP_2_ sensitivity of drTRPM2 membrane interfacial cavity mutant channels. (**A**) ADPR (100 µM, black bar) induced currents of wild-type and mutant drTRPM2 channels. Current trace of R934A (II) (**B**) and R1092A (IV) (**C**) perfused with increasing concentrations of PIP_2_ (green bars). Polylysine (15 µg/mL, PL, orange bar) was used to scavenge PIP_2_ since even in the absence of external PIP_2_ the channels are already saturated with natural membrane PIP_2_. (**D**) Summary of the presented data showing a right shift in PIP_2_ sensitivity of mutants R934A (II) and R1092A (IV). Data represented as mean ± SEM; *n* = 5–6.

To evaluate whether the decreased current was due to decreased PIP_2_ sensitivity, we employed dose-response measurements and determined EC_50_ values for PIP_2_. PIP_2_ EC_50_ for hsTRPM2 K918A (II) and R1077A (IV) were 52.2 µM and 36.3 µM respectively, displaying a pronounced rightward shift when compared with wild type hsTRPM2 (11.9 µM) (Figure 5B–D). Therefore, K918 (II) and R1077 (IV) seem to be crucial for PIP_2_ interaction, and removal of either residue causes substantially reduced PIP_2_ sensitivity. Next, we tested the corresponding residues in drTRPM2. W718A (I) and R1082A (III) did not show any currents in response to the application of ADPR (Figure 7A).

**Figure 8:**
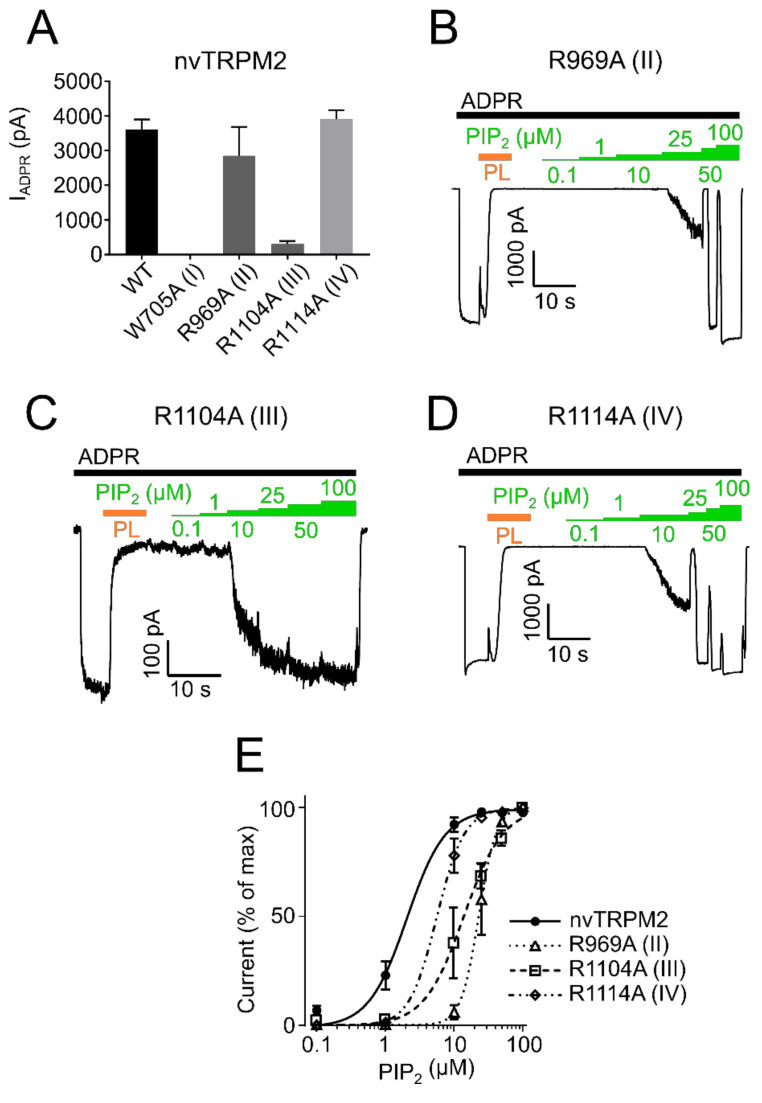
PIP_2_ sensitivity of nvTRPM2 membrane interfacial cavity mutant channels. (**A**) Current amplitudes induced by ADPR (100 µM, black bar) of wild-type and mutant nvTRPM2 channels. PIP_2_ (green bars) dose-response recordings of nvTRPM2 R969A (II) (**B**), R1104A (III) (**C**) and R1114A (IV) (**D**) in presence of 100 µM ADPR (black bar). Since maximum currents were already observed in absence of external PIP_2_, natural PIP_2_ was scavenged via polylysine (15 µg/mL, PL, orange bar) and subsequently restored via increasing PIP_2_ concentrations. (**E**) Data summary of PIP_2_ sensitivity of mutants R969A (II), R1104A (III), and R1114A (IV) showing dominant right shift compared with wild-type nvTRPM2. Data represented as mean ± SEM; *n* = 5–6.

However, since surface expression was increased compared to wild type (Figure 6) the mutants appear to be non-functional and were not further studied. R934A (II) and R1092A (IV) showed robust currents with a slightly higher current amplitude compared with WT drTRPM2 (Figure 7A). Both mutants displayed decreases in sensitivity to PIP_2_, that trended towards rightward shifts of the EC_50_ values with 34.6 µM for R934A (II) and 33.6 µM for R1092A (IV) but were not statistically significant when compared with wild type drTRPM2 (22.3 µM) (Figure 7B–D). For nvTRPM2 W705A (I) no currents were observed in the presence of ADPR similar to a mutation I in hsTRPM2 and drTRPM2 (Figure 8A). Even though surface expression for W705A (I) is reduced but still prominent, the mutant was considered non-functional and not subjected to further investigations (Figure 6). R1104A (III) displayed minuscule current amplitudes while R969A (II) and R1114A (IV) showed ADPR induced currents comparable to wild-type nvTRPM2 (Figure 8A). Analysing PIP_2_ sensitivity of the three functional mutants shows a distinct right shift with R969A (II) displaying the most prominent effect (Figure 8B–E). The EC_50_ for R969A (II), R1104A (III), and R1114A (IV) was 22.9 µM, 14.2 µM, and 5.6 µM respectively while the wild type was 2.1 µM (Figure 8E). Please note that the PIP_2_ dose-response curves in all three channel orthologues do not saturate in all mutants and EC_50_ values might be even larger than stated in this study.

These data suggest that positively charged amino acids within the membrane interfacial cavity are important for PIP_2_ activation and potentially facilitate PIP_2_ interaction within the channel. Interestingly, mutation I (conserved tryptophan within the MHR4-PreS1 domain) causes loss of function in all three channel orthologues indicating the great importance of this residue. Even though this could potentially indicate the significance of this residue for PIP_2_ interaction and therefore channel function, the loss of function could also be explained by protein misfolding or conformational changes. In addition, mutation II within the S4–S5 linker region had the strongest effects on PIP_2_ sensitivity in all three channel orthologues and could therefore represent a key residue for PIP_2_ interaction.

## 3. Discussion

In this study, we investigated the dependence of the TRPM2 function on PIP_2_. Owing to pronounced species-specific variability in TRPM2 gating we investigated three-channel orthologues of TRPM2 (human, zebrafish, and sea anemone) heterologously expressed in mammalian HEK-293 cells via inside-out patch-clamp recordings. As the main finding, we demonstrated that PIP_2_ essentially contributes to ADPR-induced activation of TRPM2 in all three species. However, there were remarkable differences in the extent to which currents could be restored after PIP_2_ depletion. In hsTRPM2, there was no restoration at all unless intracellular Ca^2+^ was supplemented in high concentrations along with PIP_2_. Our results indicate that PIP_2_ interacts with residues within the membrane interfacial cavity of TRPM2 and facilitates channel activation in the presence of ADPR and Ca^2+^.

### 3.1. PIP_2_ Is a Necessary Cofactor for TRPM2 Function

PIP_2_ dependence of hsTRPM2 and nvTRPM2 expressed in *Xenopus* oocytes was demonstrated previously showing TRPM2 currents suppressed by polylysine application and fully reactivated via PIP_2_ [30,31]. In the mammalian expression system employed in the present study, removal of endogenous PIP_2_ by polylysine followed by exogenous PIP_2_ application was not sufficient for a re-activation of hsTRPM2 even at high PIP_2_ concentrations (100 µM). The most likely explanation for the discrepancy in our opinion is the huge differences in free Ca^2+^. In fact, increasing intracellular free Ca^2+^ from 1 µM to non-physiological concentrations of 100 µM results in a significant current recovery in hsTRPM2 via PIP_2_. In contrast, nvTRPM2 was able to fully and rapidly restore currents via exogenously applied PIP_2_ and physiological calcium concentrations (1 µM), whereas drTRPM2 only recovered partially. This may be explained by the increased calcium sensitivity and conductance of nvTRPM2 compared with hsTRPM2 [23,31]. In this scenario, physiological calcium concentrations in combination with PIP_2_ are sufficient to reactivate the channel whereas hsTRPM2 requires higher calcium concentrations for reactivation. This seems plausible as it was suggested that PIP_2_ and calcium may stabilize the open state of nvTRPM2 independently [31] and therefore both calcium and PIP_2_ may be required in sufficient amounts to support the reactivation of TRPM2 channels. Alternatively, it was proposed that the PIP_2_ head group itself may be involved in calcium-binding of nvTRPM2 [31] which also highlights the co-dependent action of calcium and PIP_2_ in activating TRPM2. Moreover, endogenous PIP_2_ concentrations may be insufficient to fully activate hsTRPM2, especially during stimulation under moderate Ca^2+^ concentrations, since the application of PIP_2_ further increases currents in hsTRPM2. Interestingly, nvTRPM2 and drTRPM2 already displayed maximal currents at endogenous PIP_2_ levels within the membrane patch, which was not further increased by additional PIP_2_ application. However, it should be noted that using lower ADPR and Ca^2+^ concentrations when stimulating drTRPM2 and nvTRPM2 could result in reduced starting open probabilities which may result in further stimulation upon PIP_2_ addition.

Therefore, hsTRPM2 might be more susceptible to regulation by changes in intracellular PIP_2_ concentrations, while nvTRPM2 might be saturated at most times due to its high PIP_2_ sensitivity and therefore less susceptible to changes at endogenous PIP_2_ levels. DrTRPM2 seems to be right in between those two extremes since it is fully activated at endogenous PIP_2_ levels but is unable to fully recover after PIP_2_ removal via polylysine.

### 3.2. Structural Elements of TRPM2 for PIP_2_ Interaction

Interaction of PIP_2_ with ion channels is believed to rely on one of two different models or a combination of both [36]. The first model proposes more than 10 basic amino acids in close proximity by sequence generating a strong electrostatic site for nonspecific interactions with negatively charged head groups of phosphoinositides as is the case for the basic effector domain (BED) in MARCKS (myristoylated alanine-rich C kinase substrate). The second model is based on the pleckstrin homology domain (PH domain) in e.g., PLCδ1 (phospholipase-Cδ1) which represents multiple basic amino acids that are not adjacent to one another within the amino acid sequence but are part of a binding pocket within the folded protein. Therefore, BEDs represent unspecific electrostatic binding areas for phosphoinositides while PH domains represent a more specific binding pocket in addition to the electrostatic forces of the basic amino acids [36]. As a result, BEDs bind various phosphoinositides due to their low specificity whereas PH domains are more likely to bind to a specific phosphoinositide for steric reasons. In TRPM2, a cluster of basic amino acids in the primary sequence is absent. Therefore, PIP_2_ binding of TRPM2 may be facilitated via basic amino acids as part of a binding pocket within the folded protein even though phosphoinositide specificity is not limited to PI(4,5)P_2_ alone as PI(3,4,5)P_3_ and in part PI(3,4)P_2_ (drTRPM2) restore channel activity. Our finding that basic amino acids within the membrane interfacial cavity are necessary for PIP_2_ interaction is in agreement with this scenario. This is supported by a cryo-electron microscopy study of nvTRPM2 showing a phospholipid density within the membrane interfacial cavity [31]. In addition, this area represents a conserved region, as the binding site of PIP_2_ in TRPM8 is located in a similar site and PIP_2_ binding is facilitated by conserved residues [27,32,33]. It should be noted that the corresponding residue for mutant IV in TRPM8 does not directly interact with PIP_2_ [32] even though mutation of this residue caused the strongest rightward shift of PIP_2_ EC_50_ in TRPM8 [33] and may therefore affect activation by PIP_2_ indirectly. Furthermore, we cannot exclude that other residues examined in this study (e.g., mutant I and III in hsM2 and drM2), may also be involved in PIP_2_ binding, as the loss of function in these mutants may suggest a crucial role in PIP_2_ coordination or impairment of the channel protein by other mechanisms (e.g., misfolding). Interestingly, the two corresponding residues in TRPM8 coordinate direct binding between pre-S1 and TRP-like domains mediating gating and functional regulation by PIP_2_ [27]. Therefore, loss of function in hsTRPM2 and drTRPM2 of mutants I and III may be a result of a missing N-C interaction facilitated via PIP_2_. Moreover, mutant III could also represent a key residue for PIP_2_ binding. This is because mutation of this basic residue may disrupt PIP_2_ binding completely in hsTRPM2 and drTRPM2 due to their lower PIP_2_ sensitivity resulting in loss of function of the channel, whereas in nvTRPM2 PIP_2_ binding via the remaining residues may still be possible due to the strong PIP_2_ sensitivity of nvTRPM2. Finally, we cannot rule out potential further residues involved in PIP_2_ binding that have not been investigated in this study or that the mutations performed in this study may also affect ADPR and or Ca^2+^ responses. Future studies investigating the co-structure of TRPM2 in complex with PIP_2_ will likely provide definitive proof for the PIP_2_ binding site in TRPM2.

### 3.3. Physiological Role of PIP_2_ Interaction with TRPM2

PIP_2_ is a minor but specific component of the plasma membrane in eukaryotic cells [37]. This allows ion channels that are specifically activated by PIP_2_ to remain inactive while embedded in intracellular membranes during synthesis and transport to the plasma membrane [37,38]. This feature may also be true for TRPM2 as this channel requires PIP_2_ for full activation. In addition, PIP_2_ may act as a modulator of TRPM2 activity in diverse physiological processes. Therefore, TRPM2 activity may be modulated by changes in PIP_2_ levels within the plasma membrane. This may be achieved by activation of receptors coupled to lipid kinases or PLC resulting in transient changes of specific membrane lipids sufficient to modulate TRPM2 function. This is of particular interest for ion channels with low PIP_2_ affinity as the membrane concentration of lipids is probably changed by less than one order of magnitude during receptor activation of enzymes [38]. Therefore, hsTRPM2 with its low PIP_2_ affinity might be more susceptible to such regulatory changes compared with drTRPM2 while nvTRPM2 may not be regulated by changes in PIP_2_ levels due to its significantly higher affinity. In hsTRPM2, depletion of PIP_2_ may stop the positive feedback loop of excessive Ca^2+^ influx. As a result of activation of G protein-coupled receptors (GPCR), PLC cleaves PIP_2_ into IP_3_ and diacylglycerol, reducing Ca^2+^ influx through TRPM2 during increased Ca^2+^ elevation as a result of IP_3_-induced Ca^2+^ release from internal storages [39]. It is also worth mentioning that TRPM2 has been implicated in Alzheimer’s disease [40,41]. Interestingly, PLC is increased and accumulates in the brains of Alzheimer’s disease patients which aligns well with a report of reduced levels of phosphoinositide in these patients [42,43,44]. Therefore, it would be of great interest to test the influence of TRPM2 regulation via phosphoinositides in Alzheimer’s disease.

In conclusion, we have demonstrated that PIP_2_ enhances TRPM2 activity in all three species by means of homologous, highly conserved structural elements. The gradual requirement for the phospholipid varies remarkably between the channel orthologues, however, the uniform principle strongly contrasts the divergence that has been shown for the mechanisms used by ADPR when it gates TRPM2 in various species. The study contributes to our evolving understanding of complex modulatory processes in TRPM2 channel activation and their structural basis. These may have developed during the evolution of TRPM2 to fulfil divergent functional roles in different species and habitats.

## 4. Materials and Methods

### 4.1. Molecular Biology

Subcloning of the TRPM2 cDNA from human, zebrafish (*Danio rerio*), and sea anemone (*N. vectensis*) into the modified pIRES-hrGFP-2a vector was described previously [13,23]. Site-directed mutagenesis was performed via the Quikchange mutagenesis kit according to the manufacturer’s instructions (Agilent, Santa Clara, CA, USA), and sequences were verified (MWG-Biotech, Ebersberg, Germany). Custom-made oligonucleotides were acquired from MWG-Biotech. For immunohistological detection in surface expression experiments, wild-type and mutant channels were C-terminally fused with a triple hemagglutinin-tag (3×HA-tag) as previously described [16].

### 4.2. Biotinylation

Biotinylation assays were performed using the Pierce Cell Surface Protein Insolation Kit according to the manufacturer’s instructions (Thermo Fisher Scientific, Dreieich, Germany). In brief, transfected cells were grown to about 90% confluency and subsequently biotinylated and lysed. Samples (600 µg) were then incubated with NeutrAvidin beads while a small aliquot of total cell lysate was kept as a control. Elution was performed using SDS sample buffer and subjected to SDS-PAGE and Western blot analysis. β-actin was used as a control to rule out biotinylation of cytosolic proteins after cell damage. β-actin was detected via primary mouse-anti-β-actin antibody (1:1000, Cell Signaling, Frankfurt, Germany) and rabbit-anti-mouse-HRP conjugated secondary antibody (1:1000, Agilent). HA-tagged TRPM2 protein was detected via primary monoclonal-mouse-anti-HA antibody (1:1000, Sigma-Aldrich, Darmstadt, Germany) and rabbit-anti-mouse-HRP conjugated secondary antibody (1:1000, Agilent). Detection was visualized using the enhanced chemiluminescence detection system (ECL, Amersham Bioscience, Freiburg, Germany).

### 4.3. Cell Culture and Transfection

Human embryonic kidney (HEK-293) cells were purchased from the German Collection of Microorganisms and Cell Cultures. Cell culture was carried out in DMEM media (Biochrome, Berlin, Germany) supplemented with 4 mM L-glutamine, 2 mM sodium pyruvate, and 10% (*v*/*v*) foetal calf serum (Biochrome). Wild-type or mutant TRPM2 channels were heterologously expressed in HEK-293 cells after transient transfection of the corresponding cDNA using the FuGene 6 transfection reagent (Promega, Walldorf, Germany) according to the manufacturer’s protocol. The transfected cells were incubated for 24 h at 37 °C and 5% CO_2_. Afterwards, the cells were harvested for biotinylation assay and Western blot analysis. Alternatively, the cells were seeded on cell culture dishes at a suitable dilution and further incubated for 3–4 h. Then, patch-clamp experiments were performed with cells visibly positive for EGFP-expression.

### 4.4. Patch Clamp

Inside-Out Patch Clamp recordings were performed at room temperature and membrane potential of −60 mV using an Axopatch 200B amplifier and digitised via a Digidata 1440 A. Data were recorded on a personal computer with Clampex 10.7 and analysed using Clampfit 10.7 (Axon Instruments, Molecular Devices, San Jose, CA, USA). The standard bath solution contained (in mM) 145 CsCl, 8 NaCl, 2 MgCl_2_, 10 HEPES, pH 7.2 (CsOH) and the Ca^2+^ concentration was adjusted to 1 µM (0.886 mM Ca^2+^, 1 mM Cs-EGTA) or 100 µM (1.1 mM Ca^2+^, 1 mM Cs-EGTA). The pipette solution contained (in mM) 140 NaCl, 1.2 MgCl_2_, 1.2 CaCl_2_, 5 KCl, 10 HEPES, pH 7.4. For the application of ADPR, phosphoinositides, and polylysine a rapid solution exchange was performed via an RSC-200 (Bio-Logic Science Instruments, Göttingen, Germany). Patch pipettes were made of borosilicate glass (Hilgenberg, Malsfeld, Germany) and had tip resistances between 2 and 4 MΩ. A gap-free acquisition mode was used with analogous filtering at 2 kHz. ADPR (#A0752, Sigma-Aldrich, Darmstadt, Germany) and poly-L-lysine hydrobromide (#P2636, Sigma-Aldrich, Darmstadt, Germany) were diluted into the bath solution from 100 mM and 15 µg/mL aqueous stock respectively. 100 µM ADPR was used for activation of drTRPM2 and nvTRPM2 while 300 µM ADPR was used for hsTRPM2. Phosphoinositides (PI(4)P #10007711, PI(3,4)P_2_ #10008400, PI(4,5)P_2_ #64910, PI(3,4,5)P_3_ #10007764; Cayman Chemical, Tallinn, Estonia) were diluted into the bath solution from a 2.5 mM aqueous stock.

### 4.5. Data Analysis

Data are expressed as mean ± standard error of the mean (SEM). The numbers of experiments are presented as n. Graphs were generated via GraphPad Prism 6 and Inkscape 0.92. PIP_2_ dose-response recordings were calculated as a percentage of the maximum current obtained at 100 µM PIP_2_. Statistical comparisons were made using one-way ANOVA with Dunn’s multiple comparison test and paired Student’s *t*-test and differences were considered significant at * *p* < 0.05, ** *p* < 0.01, ns = not significant.

## Data Availability

The data presented in this study are available on reasonable request from the corresponding author.

## Supplementary Materials

The following are available online at https://www.mdpi.com/article/10.3390/ijms22094637/s1, Figure S1: PIP_2_ and calcium effects on hsTRPM2 and drTRPM2 activity. Figure S2: ADPR dose-response and current rundown of hs-, dr- and nvTRPM2.
