# Supplementary material for: Species-Specific Regulation of TRPM2 by PI(4,5)P2 via the Membrane Interfacial Cavity
Source: Int J Mol Sci. 2021 Apr 28;22(9):4637. doi: 10.3390/ijms22094637 (PMC8125603; doi:10.3390/ijms22094637)
Supplement: Supplementary file 1 [file ijms-22-04637-s001.zip › ijms-1156133-supplementary.pdf]

International Journal of
Molecular Sciences

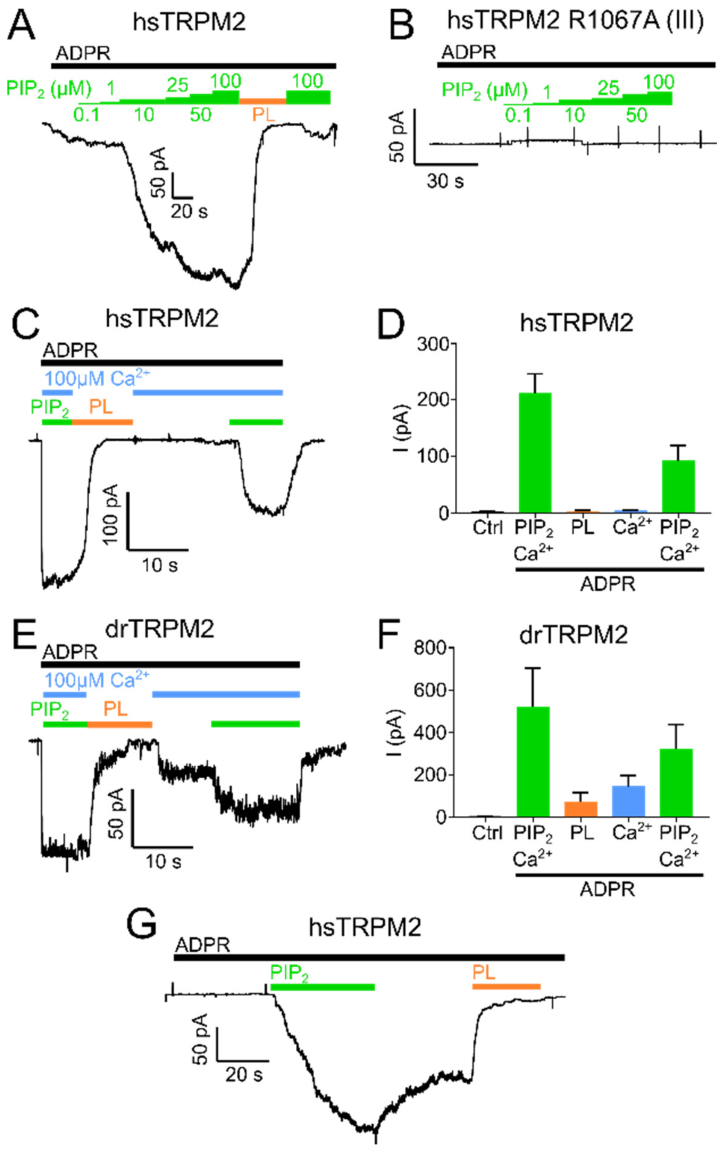

Figure S1: PIP_2 and calcium effects on hsTRPM2 and drTRPM2 activity. PIP_2 Dose-response recordings of inside-out patches of HEK-293 cells expressing hsTRPM2 (A) hsTRPM2 R1067A (III) (B). ADPR (black bar) was used to initiate TRPM2 currents followed by increasing concentrations of PIP_2 (green bars). Polylysine (PL 15µg/ml, orange bar) was utilized to scavenge PIP_2. Representative current traces and statistics of hsTRPM2 (C, D) and drTRPM2 (E, F) using elevated calcium concentrations (100µM) alone or in combination with 25 µM PIP_2 in the continued presence of ADPR (black bar). In hsTRPM2 (C, D) 100 µM calcium was not able to rescue currents after polylysine application (PL, orange bar) while drTRPM2 (E, F) showed small current recovery. Additional application of PIP_2 resulted in improved current rescue in hsTRPM2 and drTRPM2. (G) Effect of PIP_2 washout on hsTRPM2 currents (300µM ADPR, 25µM PIP_2). Data represented as mean ± SEM; n = 4 - 5.

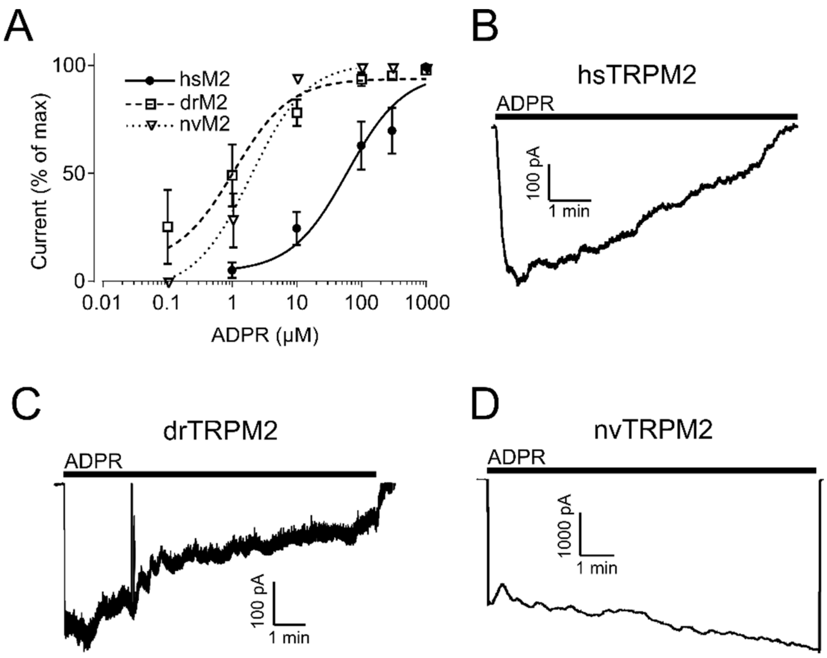

Figure S2: ADPR dose-response and current rundown of hs-, dr- and nvTRPM2. (A) Statistics of ADPR dose-response recordings of inside-out patches of HEK-293 cells expressing hsTRPM2, drTRPM2 and nvTRPM2 using 1 µM intracellular Ca^{2+}. ADPR EC_{50} was 60.9 µM for hsTRPM2, 1.1 µM for drTRPM2 and 1.8 µM for nvTRPM2 (n = 4-5). Representative traces of current rundown for hsTRPM2 (B), drTRPM2 (C) and nvTRPM2 (D) using 1 µM intracellular Ca^{2+} and 300 µM ADPR (hsTRPM2) and 100µM ADPR (drTRPM2 and nvTRPM2).
